# Complex and lasting impacts of heatwaves on life-history traits and fitness in *Daphnia magna*

**DOI:** 10.1242/jeb.250837

**Published:** 2025-09-26

**Authors:** Jie Xiao, Wen-Xiong Wang

**Affiliations:** ^1^ School of Energy and Environment and State Key Laboratory of Marine Environmental Health, City University of Hong Kong, Kowloon, Hong Kong, China; ^2^ Research Centre for the Oceans and Human Health, City University of Hong Kong Shenzhen Research Institute, Shenzhen 518057, China

**Keywords:** Climate change, Extreme temperature event, Phenotypic Plasticity, Warming, Resilience

## Abstract

Rapid climatic fluctuations, such as heatwaves, are key drivers of ecological disruption and pose significant physiological challenges to ectothermic organisms, yet their capacity for short- or long-term adaptation and transgenerational effects remain poorly understood. Using the model freshwater zooplankton *Daphnia magna*, we experimentally tested the physiological resilience, acclimation and evolutionary responses in *D. magna* across multiple generations under simulated heatwave conditions. Heatwaves significantly compromised the development and reproduction function of *D. magna*, with detrimental effects amplified under low food availability. Moreover, general temperature responses (stable warming) failed to predict heatwave responses, even within the same temperature range. This appears to be largely due to the complex interplay of warming and cooling effects during heatwaves, where the cooling phase within heatwaves unexpectedly elicited physiological disruption, highlighting overlooked complexity in heatwave dynamics. We found no evidence of short-term acclimation to heatwaves, whereas the impact of a second heatwave was additive or even multiplicative. Molecular biomarker profiles, coupled with physiological responses, revealed transgenerational impacts of heatwave exposure in *D. magna* and pointed to a potential trade-off between life-history investment and heat tolerance. Despite repeated exposure to multigenerational and periodic heatwaves, *D. magna* populations may still struggle to develop lasting heatwave tolerance. These findings underscore the physiological complexity of heatwave responses and raise concerns about the adaptive potential of aquatic ectotherms facing increasingly variable thermal regimes.

## Teaser

**Summary:** Responses of *Daphnia magna* to heatwaves are not predictable from responses to stable warming; cooling phases and limited food availability exacerbate the negative impacts of heatwaves, with evidence of transgenerational effects.

## INTRODUCTION

Climate change is profoundly affecting biota on a global scale, disrupting ecosystems and their functions worldwide (Brierley and Kingsford, 2009; Hoegh-Guldberg and Brubo, 2010; Malhi et al., 2020). The increasing frequency, intensity and duration of extreme events, such as heatwaves, are significantly altering ecosystem structure and biodiversity, with the trends expected to intensify under ongoing global warming (Smale et al., 2019; Wernberg et al., 2013). Heatwaves, defined as prolonged periods of excessive heat, are a specific type of extreme temperature event (Perkins and Alexander, 2013). A more commonly used definition describes a heatwave as a period when the daily temperature maximum exceeds the local average maximum by 5°C for more than five consecutive days (Hobday et al., 2016). These events are rapidly emerging as powerful ecological disturbances, with the potential to restructure entire ecosystems and disrupt the provision of ecological goods and services lasting throughout the 21st century (Frölicher et al., 2018; Oliver et al., 2018). To date, general temperature effects on genetic changes (Bradshaw and Holzapfel, 2008; Brans et al., 2017), as well as alterations in physiology (Lefevre et al., 2021), morphology (Ryding et al., 2021), behavior (Nagelkerken and Munday, 2016) and life-history traits (Adrian et al., 2006) have been well described. However, relatively few studies have examined the roles of evolutionary adaptation and phenotypic or transgenerational plasticity in coping with heatwave stress, limiting our ability to predict future ecological responses. To address this gap, we employed an experimental, multigenerational approach to investigate both plastic and evolutionary responses to heatwave stress in a model zooplankton system.

Phenotypic plasticity is the ability of a single genotype to express different phenotypes in response to environmental conditions (Via and Lande, 1985). Adaptive or non-adaptive plasticity depends on whether environmentally induced phenotypes take an organism closer to or further from the local optimum (Ghalambor et al., 2007). Some organisms rely on phenotypic plasticity to persist and adapt to fluctuating environmental conditions, which can enhance fitness and population viability (Chevin and Hoffmann, 2017; Reed et al., 2010). This has been demonstrated for behavioral (Caspi et al., 2022), morphological (Cooper et al., 2022) and life-history traits (Stearns and Koella, 1986). Phenotypic variation plays a role in heat stress responses in a broad range of taxa (Kelly et al., 2017; Mallard et al., 2020; Pereira et al., 2017; Valluru et al., 2017). Broadly, most research on adaptive plasticity has focused on single-generation responses, often using common garden experiments to control for direct environmental effects. While such studies suggest that phenotypic variation in thermal tolerance may have a heritable component (Sasaki and Dam, 2019; Schaum et al., 2022), they primarily capture plastic responses within a single generation, leaving transgenerational plasticity in response to heatwaves largely unexplored. Transgenerational plasticity occurs when environmental experiences or stressors encountered by one generation influence the phenotype, behavior or physiology of subsequent generations, even in the absence of direct exposure (Donelson et al., 2018; Ho and Burggren, 2010; Shama and Wegner, 2014). Understanding transgenerational plasticity is crucial for predicting how organisms may cope with rapidly changing environments over multiple generations (Bell and Hellmann, 2019). However, the extent, persistence and adaptive significance of transgenerational responses to heatwaves remain poorly understood, particularly in the context of climate change.

While thermal adaptation under stable warming has been well studied, much less is known about the capacity of organisms to tolerate recurrent heatwaves, which are more abrupt and variable. In response to climate change, species may shift their distributions, adapt to new environments or acclimate through phenotypic plasticity, enabling them to cope with rapidly changing conditions (Donelson et al., 2018; Hoffmann and Sgrò, 2011; Munday et al., 2013). Plasticity is likely to be particularly important because the potential for rapid genetic adaptation may be limited under predicted climate change scenarios (Donelson et al., 2018; Merilä, 2012). One important form of plasticity is transgenerational plasticity, in which environmental conditions experienced by parents influence offspring phenotypes, thereby mitigating the impacts of environmental change and buffering populations against climate stress (Chevin et al., 2010; Guillaume et al., 2016; Yin et al., 2019). Such parental effects can also be mediated by epigenetic mechanisms, including DNA methylation, histone modifications and small RNAs, that alter gene expression and can be transmitted across generations independently of the underlying DNA sequence (Ho and Burggren, 2010). These process can be influenced by the environment and thus provide a mechanism by which the parental environment can influence the performance of offspring (Donelson et al., 2018). Accordingly, previous studies have shown that exposure to elevated temperatures can induce transgenerational effects on growth, reproduction and survival (Donelson et al., 2016; Salinas and Munch, 2012; Walsh et al., 2014). Despite this, it remains unclear whether populations can sustain their fitness under recurrent heatwaves through transgenerational plasticity when exposed across multiple generations.

In this study, we conducted experiments using the water flea *Daphnia magna*, a keystone branchiopod crustacean widely used as a model species in ecology, evolution and ecotoxicology (Abdullahi et al., 2022). Several key traits of *D. magna*, including its short generation time, parthenogenetic reproductive cycle and high potential for rapid evolution, make it an ideal model for studying heatwave responses. *Daphnia magna* inhabits a diverse range of ecological environments, from freshwater to brackish water, and plays a crucial role in aquatic food webs, making it particularly vulnerable to climate change (Altshuler et al., 2011; Miner et al., 2012). Despite increasing empirical evidence on the effects of heatwaves on *D. magna*, including physiology, survival and reproduction (Nash et al., 2022; Müller et al., 2018; Nowakoski and Slugocki, 2021), less is known about the transgenerational and multigenerational impacts of episodic heatwaves, particularly how repeated exposure across generations affects tolerance and fitness. Leveraging the biological and ecological characteristics of *D. magna*, we aimed to address the following questions. (1) Can plastic responses to general elevated temperatures (stable warming) predict responses to heatwaves of the same temperature range in *D. magna*? (2) What are the transgenerational impacts of heatwaves and stable warming? (3) Does short-term acclimation to heatwaves enhance resistance to subsequent heatwave exposures? (4) Can *D. magna* populations develop tolerance to heatwaves through multigenerational exposure? We specifically hypothesize that, as a specific type of extreme temperature event, heatwaves may impose stronger negative effects on *D. magna* than stable warming of the same temperature range, because of their complex and fluctuating nature. Moreover, these detrimental effects may carry over across generations, thereby reducing fitness in offspring even when they are not directly exposed.

To address these questions, we experimentally exposed *D. magna* neonates to a 5 day heatwave (20°C to 26°C) and a stable warming treatment (constant 26°C), with 20°C serving as the control. Both temperatures are within a range reasonably close to the optimal thermal conditions for *D. magna* growth. We assessed phenotypic plasticity by measuring life-history traits under stable warming and heatwave conditions and examined the effects of repeated heatwave exposure on reproductive performance. To investigate transgenerational plasticity, we exposed maternal *D. magna* to heatwaves and assessed the effects on offspring development and reproduction. Finally, we subjected *D. magna* (from F0 to F4) to repeated heatwaves to evaluate the evolutionary potential for plastic responses to heatwave tolerance. This experimental design enables us to disentangle the relative importance of phenotypic and transgenerational plasticity in zooplankton responses to heatwaves and stable warming. Furthermore, by studying the implications of heatwave scenarios for the crucial biological trait of population viability, our study provides critical insights for predicting the ecological consequences of heatwaves in the context of global climate change.

## MATERIALS AND METHODS

### *Daphnia magna* culture

*Daphnia magna* Straus 1820 was obtained from our permanent laboratory culture, maintained for approximately 20 years. The culture medium used throughout the experiment was SM7 medium as previously described (Yan et al., 2018). The animal culture and experimental procedures followed OECD guidelines. At the start of the experiment, a single clonal lineage of *D. magna* was cultured under controlled laboratory conditions (16 h:8 h light:dark photoperiod, 20°C) for four generations to minimize maternal effects. The animals were fed *Chlamydomonas reinhardtii* at a concentration of 5×10^4^ cells ml^−1^ daily, and 80% of the medium was refreshed twice per week throughout the experiments. *Daphnia* species are characterized by short generation times (approximately 8–11 days at 20°C) and a predominantly parthenogenetic reproductive cycle (Korpelainen, 1986). This mode of reproduction enables phenotype studies while excluding genetic variation, allowing us to distinguish genetic from non-genetic effects.

### Experimental design

Synchronized *D. magna* neonates (<24 h old) were used in our experiments. The experimental setup included three groups: control, heatwave (HW) and stable warming (SW). We selected a 5 day heatwave exposure period as this duration is commonly used in meteorological studies (Mazdiyasni and AghaKouchak, 2015).

At the start of Experiment 1, which tested life-history traits under different HW and SW conditions, each treatment group included six jars, with five female neonates per 100 ml jar, resulting in six biological replicates per group (*n*=6). Body length (measured from the top of the eye to the base of the tail spine) was recorded for 10 neonates at the beginning of the experiment using an optical microscope (Nikon Eclipse Ti2 Inverted Microscope). The neonates were then randomly assigned to one of three temperature treatments: control (20±0.2°C), HW (26±0.2°C) or SW (26±0.2°C). The temperature was achieved by a thermostat-controlled heating pad. After 5 days of heatwave exposure, the temperature for the HW group was gradually reduced from 26 to 20°C at a rate of 2°C per hour to minimize temperature stress. Water temperature was monitored three times daily, with fluctuations limited to ±0.2°C. Life-history traits were assessed for each group. Size at maturity was recorded when individuals first carried a brood in the brood pouch. We quantified average clutch size (first and second broods), time to reproduction, total number of broods and total offspring produced by day 21. The somatic growth rate, defined as (size at maturity−size at birth)/time to maturity (mm day^−1^), was additionally calculated. To assess the potential effects of the cooling phase during a heatwave on *D. magna*, we additionally conducted a cooling experiment where the temperature was gradually reduced from 26°C to 23°C, resulting in a fluctuation of only 3°C (defined as HW3°C group). Because of potential variability in cohort synchronization, it may be hard to differentiate between first and second clutch release across all six jars. We randomly selected two individuals from each jar (with the same label) and transferred them to new 100 ml jars (one per jar) for daily offspring monitoring. New neonates were removed and placed in separate jars daily. No dormant eggs were observed throughout the 21 day experiment.

In Experiment 2, we aimed to determine the impact of additional heatwaves on reproductive output. Experimental *D. magna* were cultured under the same conditions as described in Experiment 1. We established three groups: control, first heatwave (1st HW) and second heatwave (2nd HW). Each group was maintained in 100 ml jars, with five female neonates per jar and six replicates per group. Experimental jars in the treatment groups were exposed to heatwave conditions. In the 2nd HW group, individuals experienced an initial 5 day heatwave, followed by an additional 5 day heatwave on day 10 after a brief acclimatization period. On day 15, they were returned to 20°C for the remaining 6 days of the experiment. As our focus was on overall reproductive output, we quantified the total average number of offspring per jar over 21 days.

In Experiment 3, we aimed to investigate the influence of food availability on the growth of *D. magna*. As before, the experiment was conducted using 100 ml jars, each inoculated with five female neonates, with six replicates per generational experiment. We set up two groups, including control (food concentration: 5×10^4^ cells ml^−1^ per day) and low food group (food concentration: 5×10^2^ cells ml^−1^ per day). Throughout the experiment, low food concentration had a large effect on the development and growth and survival of *D. magna*, so we measured the body length of *D. magna* and recorded the mortality rate after heatwave on the 6th day.

In Experiment 4, we aimed to investigate the transgenerational plasticity of *D. magna* in response to heatwave and warming across multiple generations. The overall experimental design was similar to that of Experiment 1, but we additionally observed life-history trait changes over four generations. The experiment was conducted using 100 ml jars, each inoculated with five female neonates, with six replicates per condition, consistent across generations. We established three experimental groups. In the control group, jars were maintained at 20°C across all generations. In the HW group, the F0 generation experienced a heatwave but was subsequently cultured at 20°C for the following four generations (F1–F4). In the SW group, jars were maintained at 26°C across all generations. To minimize the effects of brood batch differences, we used only second-brood neonates as parents for the subsequent generation.

In Experiment 5, we aimed to test whether *D. magna* populations could develop tolerance to heatwaves through evolutionary adaptation. The experiment was conducted using 100 ml jars, each inoculated with five female neonates, with six replicates per generational experiment. We performed a multigenerational heatwave exposure experiment: the F0 generation was exposed to a 5 day heatwave before being returned to normal temperature conditions (20°C). The offspring (F1) were then subjected to the same heatwave conditions for 5 days before being returned to 20°C, with this cycle continued for four consecutive generations (F4). To ensure a sufficient number of individuals for subsequent experiments, we used only second-brood neonates as parents for the next generation.

### Expression profiles of vitellogenin genes across generations

Vitellogenin genes play a critical role in the reproduction and development of *D. magna* (Tokishita et al., 2006). To investigate the expression profiles of *vtg1* and *vtg2* in response to heatwaves and warming across multiple generations, we conducted quantitative gene expression analysis. The experimental methods followed those described in Experiment 4. Samples were collected on day 6 from each group (control, HW and SW) and across five generations (F0–F4). To obtain sufficient RNA for downstream analyses, samples from two jars were pooled, resulting in three biological replicates per group. Total RNA was extracted using a commercial extraction kit (Takara Bio Inc.) following the manufacturer's instructions. cDNA was synthesized using the PrimeScript cDNA Synthesis Kit (Takara Bio Inc.). Quantitative PCR (qPCR) was performed using a 7300 RT-PCR system (Applied Biosystems), as described in a previous study (Xiao and Wang, 2022). The primers used for qPCR analysis were as follows: *vtg1*: F: CCGTCGCTGAAGTCATCTTG, R: ACAGTCTCGGAGCCAATCAA; *vtg2*: F: CATTTGAGACATGGGCAGAC, R: GCACAGTCATTACGAGAACG. Gene expression levels were calculated using the 2^−ΔΔCt^ method and normalized to β-actin (F: GCCCTCTTCCAGCCCTCATTCT, R: TGGGGCAAGGGCGGTGATTT).

### Statistical analysis

Principal component analysis (PCA) was conducted in R version 4.3.1 (https://www.r-project.org/) using the prcomp() function from the base stats package. The life-history traits were included as input variables: age at maturity, size at maturity, first clutch size, population size at day 21, somatic growth rate and time to first reproduction. All variables were standardized prior to analysis to ensure equal weighting and eliminate scale effects. There were no missing data in the dataset. All traits were measured from individual-level data across the three treatments (control, SW and HW), with six biological replicates per treatment. Data are presented as the mean±s.e.m. from at least three independent experiments. Data analysis was performed using one-way ANOVA or Student's *t*-test in SPSS software (version 20.0, SPSS Inc., Chicago, IL, USA). Assumptions of normality and homogeneity of variances were verified using Shapiro–Wilk and Brown–Forsythe tests, respectively. *P*<0.05 was considered statistically significant.

## RESULTS

### Differential impacts of heatwaves versus stable warming on life-history traits

**Fig. 1.**
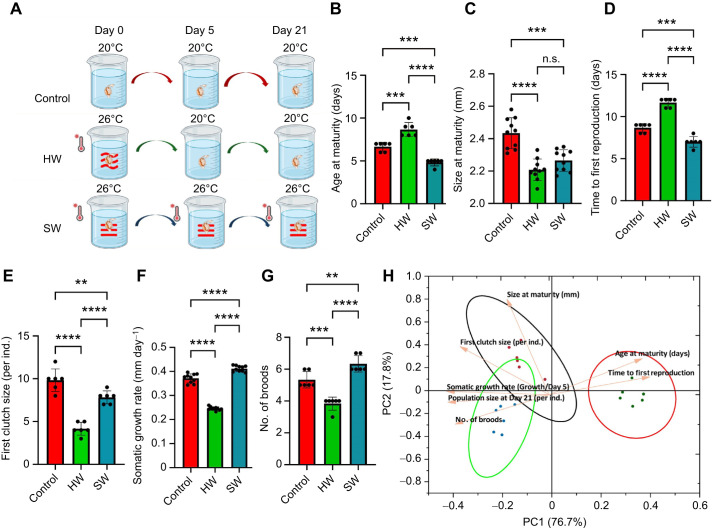
**Heatwave exposure impairs development and reproduction in *Daphnia magna*.** (A) Overview of the experimental design for Experiment 1, showing the three temperature treatments: control (20°C), heatwave (20°C to 26°C; HW) and stable warming (constant 26°C; SW). Each treatment included six independent replicates, with five individuals per replicate. (B–G) Life-history traits across the control, HW and SW treatment groups: age at maturity, size at maturity, time to first reproduction, first clutch size, somatic growth rate and population size at day 21 (number of broods), respectively. (H) Principal component analysis (PCA) showing differences among the three groups based on life-history traits. Input variables included age at maturity, size at maturity, time to first reproduction, first clutch size, somatic growth rate and population size at day 21. Variables were standardized prior to PCA to ensure equal weighting. All values are presented as means±s.e.m., with each point representing one replicate mean. Statistical comparisons were conducted using Student's *t*-test for pairwise contrasts. Asterisks indicate significance: **P<0.01, ***P<0.005, ****P<0.0001.

We employed an experimental approach to investigate how climate change affects population viability by examining the detailed impacts of heatwave and warming conditions on *D. magna* (Fig. 1A). The time to maturity was extended by 20% following heatwave treatment compared with the control, whereas stable warming resulted in a 15% reduction in time to maturity relative to both the control and heatwave treatments (Fig. 1B). However, exposure to both heatwaves and stable warming led to significant reductions in mature body size (HW: −20%, SW: −15%; Fig. 1C). Additionally, significant differences were observed among the two experimental groups across five key life-history traits. *Daphnia magna* exposed to a single heatwave exhibited a substantial decline in developmental and reproductive performance, with a greater reduction (∼60%) in reproductive output compared with individuals maintained at a stable elevated temperature of 26°C (Fig. 1D–G; Fig. S1). The PCA results further supported these distinct responses, with individuals clustering into three separate groups based on life-history traits of each thermal treatment (PC1=76.7%, PC2=17.8%; Fig. 1H; Table S1).

### Role of cooling phases in heatwave stress

**Fig. 2.**
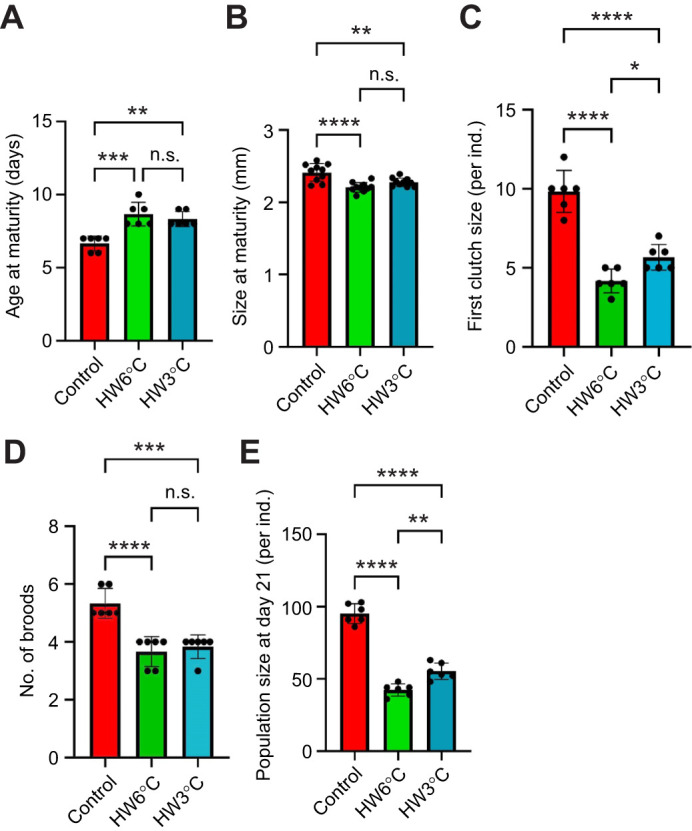
**Effects of cooling phases during heatwaves on *D. magna.*** (A–E) Life-history traits across the control and two different cooling treatments: temperature reduction of 3°C or 6°C following heatwave exposure. Each treatment consisted of six independent replicates, with five individuals per replicate. Data are presented as means±s.e.m., with each point representing one replicate mean. Statistical comparisons among treatments were performed using Student's *t*-test. Asterisks indicate significance: *P<0.05, **P<0.01, ***P<0.005, ****P<0.0001.

Considering that cooling phases during heatwaves may also play an important role in shaping life-history traits, we conducted an additional experiment with two different cooling treatments, where temperatures were gradually reduced from 26°C to 23°C (HW3°C) or 20°C (HW6°C) following heatwave exposure. The HW3°C group exhibited a 23% increase in reproductive output compared with the HW6°C group (*P*<0.05), while no significant differences were observed in size at maturity or time to maturation (Fig. 2A–E).

### Additive impacts of recurrent heatwaves and interactive effects of food limitation and heatwaves

**Fig. 3.**
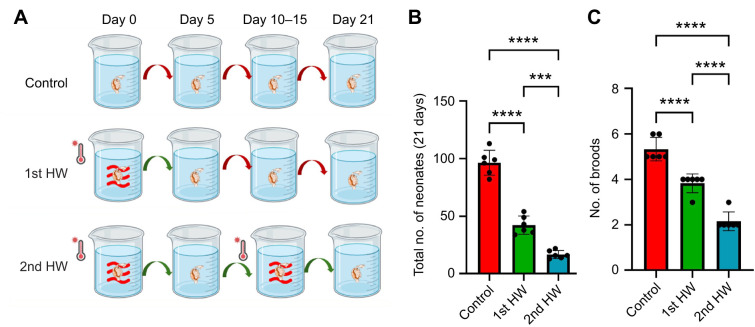
**Effects of repeated heatwaves on development and reproductive output in *D. magna*.** (A) Experimental design for Experiment 2 showing the effect of exposure to additional heatwaves, showing the three treatments: control (20°C), first heatwave (single 5 day exposure to 26°C) and second heatwave (an additional 5 day heatwave on day 10 following a brief acclimation). Each treatment consisted of six independent replicates, with five individuals per replicate. (B,C) Effects of repeated heatwaves on reproductive performance. Data are shown as means±s.e.m., with each point representing one replicate mean. Statistical comparisons among treatments were performed using Student's *t*-test. Asterisks indicate significance: ***P<0.005, ****P<0.0001.

We assessed the impact of additional heatwaves on reproductive output. *Daphnia magna* neonates were exposed to heatwave conditions for 5 days, then cultured at 20°C for 5 days of temporary acclimation followed by an additional 5 days of heatwave exposure (Fig. 3A). The results showed that the impacts of a second heatwave were additive, even after 5 days of acclimation, with the reproductive output significantly reduced (2nd HW: −60%; Fig. 3B,C). We found no evidence that short-term acclimation to heatwave stress improves resistance to subsequent heatwaves.

**Fig. 4.**
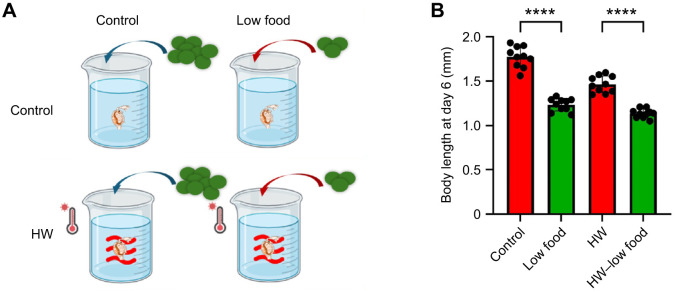
**Interactive effects of food limitation and heatwaves.** (A) Experimental design of Experiment 3 for assessing the impact of food availability, including two temperature treatments (control and heatwave) crossed with two food regimes (control food and low food). Each treatment combination consisted of six independent replicates, with five individuals per replicate. (B) Interactive effects of food limitation and heatwave exposure on individual development. Data are presented as means±s.e.m., with each point representing one replicate mean. Statistical comparisons among treatments were performed using Student's *t*-test. Asterisks indicate significance: ****P<0.0001.

To assess the interactive effects of food abundance and heatwaves on individual fitness, we exposed *D. magna* neonates to either control or heatwave conditions under low food availability and tracked their growth and development over 6 days (Fig. 4A). Our results indicated that heatwave effects on *D. magna* were food dependent. Low food availability alone significantly reduced body size (−30.4%; Fig. 4B). Under heatwave conditions, body size decreased further (−22.5%) compared with a reduction of −17.3% under normal food conditions. Notably, while neither heat stress nor low food availability alone caused significant mortality increases, their combination led to a 37% increase in mortality (HW+low food concentration; Fig. S1C).

### Transgenerational effects of heatwaves

**Fig. 5.**
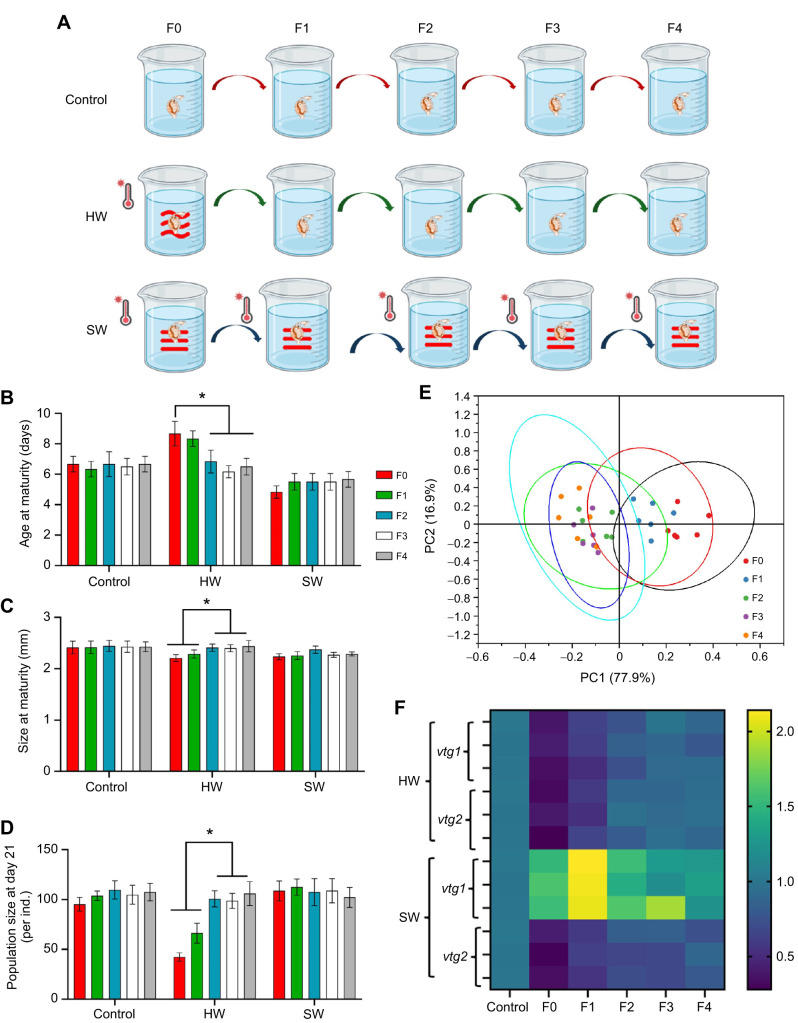
**Transgenerational effects of heatwaves on offspring fitness in *D.*** ***magna*****.** (A) Experimental design of Experiment 4 for investigating transgenerational plasticity in response to heatwave and stable warming, with three treatments: control, heatwave and stable warming. Each treatment consisted of six independent replicates, with five individuals per replicate, which were continuously observed through four subsequent generations (F1–F4). (B–D) Life-history traits across multiple generations under control, heatwave and stable warming conditions: age at maturity, size at maturity and population size at day 21, respectively. (E) PCA of life-history traits across six replicates over multiple generations, including age at maturity, size at maturity and population size at day 21. (F) Heatmap of vitellogenin gene expression (*vtg1* and *vtg2*) across treatments and generations. For qPCR analysis, samples from two jars were pooled, resulting in three biological replicates per group (*n*=3). The color scale represents fold-change values relative to control expression. All data are presented as means±s.e.m., with each point representing the replicate mean. Statistical comparisons were performed using Student's *t*-test (pairwise contrasts) and one-way ANOVA (multi-group comparisons). Asterisks indicate significance: *P<0.05.

To assess the transgenerational effects of heatwaves, we designed three experimental groups: (1) a control group maintained at 20°C across all generations (F0, F1, F2, F3 and F4), where baseline growth and reproduction were monitored in each generation; (2) a recovery group, where the F0 generation experienced a heatwave but was subsequently cultured at 20°C, allowing us to assess the persistence of transgenerational plastic effects; and (3) a stable warming group in which the temperature was maintained at 26°C across all generations (F0–F4) to evaluate potential adaptive or maladaptive plasticity under prolonged thermal stress (Fig. 5A). Life-history traits were quantified to assess the transgenerational plasticity and potential evolutionary adaptation in *D. magna*.

Our results revealed distinct transgenerational effects following heatwave exposure (Fig. 5B–D). In the F1 generation, delayed maturation, reduced body size at maturity and lower reproductive output were observed, despite the absence of direct heatwave exposure. By F2 to F4, life-history traits stabilized, with no significant differences from the control. PCA analysis further supported these findings, with F0 and F1 forming one cluster and F2–F4 forming another (PC1=77.9%, PC2=16.9%; Fig. 5E; Fig. S2, Table S2). In contrast, *D. magna* exposed to stable warming exhibited a distinct trajectory of life-history traits. Unlike the fluctuating generational effects observed under heatwave conditions, stable warming resulted in a consistent shift in life-history traits. Individuals exposed to stable warming had a shorter maturation time and reduced body size at maturity compared with controls.

Heatwave exposure significantly reduced *vtg1* and *vtg2* expression, with downregulation persisting in F1 and F2 before recovering to control levels in F3 and F4 (Fig. 5F; Fig. S3). Under stable warming, expression patterns differed: *vtg1* was significantly upregulated, whereas *vtg2* remained consistently downregulated, though with slight recovery across generations.

### Multigenerational heatwave exposure

**Fig. 6.**
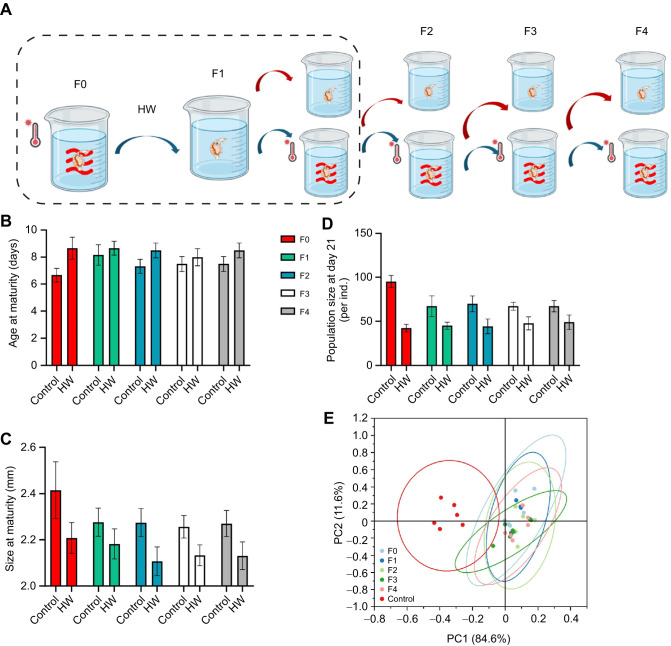
**Multigenerational assessment of heatwave tolerance in *D. magna*.** (A) Experimental setup of Experiment 5 for the heatwave selection experiment. The parental generation (F0) was exposed to a 5 day heatwave and then returned to control conditions (20°C). Offspring (F1) were either maintained continuously at 20°C (control) or exposed to the same 5 day heatwave before being returned to 20°C. This cycle was repeated over four consecutive generations (to F4). Each treatment consisted of six independent replicates, with five individuals per replicate, continuously tracked across four generations. (B–D) Life-history traits across multiple generations of *D. magna* following heatwave selection: age at maturity, size at maturity and population size at day 21, respectively. (E) PCA of life-history traits across generations after heatwave selection, including age at maturity, size at maturity and population size at day 21. All data are presented as means±s.e.m., with each point representing the replicate mean.

We conducted a multigenerational experiment (Fig. 6A), exposing the F0 generation to a 5 day heatwave (26°C) before returning them to normal temperature conditions (20°C). The offspring (F1) were then exposed to a heatwave for 5 days before being returned to 20°C, with this cycle continuing over four consecutive generations (to F4). Growth and reproductive output were tracked across all generations to assess whether *D. magna* could develop thermal tolerance through repeated exposure to short-term heatwaves or whether sustained warming was required for adaptation.

We observed no significant improvement in growth or reproductive performance despite multigenerational selection under heatwave conditions (Fig. 6B–D). Across generations, neonates from heatwave-exposed lineages exhibited a 45–55% reduction in offspring production (F1–F4) and delayed development compared with controls that had never experienced heatwaves. PCA analysis supported these findings, with all generations clustering into distinct control and heatwave groups based on life-history traits (PC1=84.6%, PC2=11.6%; Fig. 6E; Table S3, Fig. S4).

## DISCUSSION

### Heatwave responses of *D. magna* are influenced by combined impacts of warming and cooling

Our findings demonstrate that responses to heatwaves were not predictable from responses to stable warming. The more pronounced negative effects of heatwaves relative to stable warming highlighted the severity of acute, extreme temperature fluctuations on life-history traits (Salinas et al., 2019), suggesting that the physiological stress induced by transient but intense heat events may have more detrimental consequences than a sustained increase in temperature. Reduced body size and an accelerated life cycle under warming conditions are key ecological responses to climate change, often carrying fitness costs at the individual level (Lancaster et al., 2017; Verberk et al., 2021). The observed reduction in size at maturity under both heatwave and stable warming conditions, despite differences in time to maturity, is consistent with the temperature–size rule (Hoefnagel et al., 2018), a trade-off between growth rate and energy allocation under thermal stress. In heatwave-exposed *D. magna*, the delayed maturation may reflect an attempt to compensate for developmental stress, yet the reduced body size suggests that growth was constrained by the transient but extreme thermal challenge. This aligns with findings that acute thermal stress disrupts metabolic efficiency, limiting resource availability for growth (Angilletta et al., 2004).

Conversely, under stable warming conditions, the observed accelerated maturation probably reflects a proximate physiological response to elevated temperatures, consistent with temperature-dependent metabolic acceleration (Hoefnagel et al., 2018; Neuheimer and Grønkjær, 2012). The combination of a shorter maturation time and reduced body size suggests a life-history shift, where resources are prioritized for early reproduction rather than prolonged growth, which is probably an established pattern in organisms adapting to stable warming conditions (Forster et al., 2011).

Reproductive fitness was differentially impacted by heatwaves and stable warming. Heatwave-exposed *D. magna* exhibited reduced offspring production, indicating that acute heatwave stress significantly compromised reproductive function. Previous studies similarly demonstrated that extreme heatwave events impaired reproduction, with potential long-term ecological consequences for population dynamics and biodiversity loss (Bartolini et al., 2013; Sales et al., 2021). In contrast, under stable warming, the total offspring count slightly increased relative to that of the control group. This increase was accompanied by a significantly shorter time to the first brood and a greater number of reproductive events, albeit with reduced clutch sizes. These findings suggest that while stable warming may allow for physiological acclimation or adaptation, trade-offs exist between increased reproductive frequency and clutch size. Future studies need to consider not only long-term warming trends but also short-term extreme climatic events when assessing the ecological and evolutionary impacts of climate change on organisms.

The findings from our experiments suggest that cooling phases may act as additional stressors during heatwaves, rather than simply providing relief. The observed differences between the HW3°C and HW6°C groups highlight the complex interplay between warming and cooling phases during heatwaves, emphasizing the need to consider both aspects when evaluating heatwave stress responses. While low temperature stress (or cold stress) responses affecting growth, reproduction, behavior and metabolism (Garcia and Teets, 2019; Ren et al., 2020; Wen et al., 2017) have been well documented, the physiological effects of cooling phases within heatwave cycles remain largely underestimated and unexplored. Future studies on heatwave responses should therefore incorporate the effects of cooling phases (i.e. when temperatures return to baseline conditions below or above) within heatwave scenarios, as well as the specific physiological mechanisms underlying these responses.

In summary, our findings demonstrated that responses to heatwaves were not predictable from responses to stable warming. *Daphnia magna* exhibited distinct life-history shifts under each thermal scenario, underscoring the fundamentally different ecological and physiological consequences of these two climate change phenomena. Heatwaves imposed greater physiological constraints and reproductive costs, highlighting their unique and more detrimental impacts compared with stable warming. Additionally, the interplay between warm and cool phases during heatwaves warrants further investigation to fully capture the effects of fluctuating thermal environments on population fitness.

### Additive impacts of recurrent heatwaves and food limitation on fitness and population viability

The additive impacts of repeated heatwave exposure indicate that *D. magna* lacks the capacity to mount a more robust response to successive stress events. This suggests that rapid adaptation to fluctuating thermal environments may be constrained, heightening the vulnerability of populations to the increasing frequency and intensity of heatwaves predicted under climate change. Overall, our study highlights the cumulative effects of heatwaves and suggests that resilience to climate extremes may be lower than expected, raising concerns about the limits of evolutionary rescue in wild populations facing rapid and recurrent environmental stressors (Bell, 2013).

Our findings suggest that limited food availability exacerbates the negative impacts of heatwaves on *D. magna*, highlighting that environmental stressors interact synergistically rather than acting independently (Birk et al., 2020). The combination of heat stress and food limitation led to compounded reductions in growth and survival, which could threaten population viability and even drive local extinctions, particularly in populations already near ecological thresholds. Temperature–nutrient interactions are likely to be most critical during summer, when heatwaves coincide with seasonal nutrient limitations (Thomas et al., 2017). Understanding these mechanisms will be essential for identifying key drivers of population persistence and disentangling the complex interactions between climate and resource availability. As climate change continues to increase the frequency and severity of environmental fluctuations, maintaining stable food resources in managed aquatic ecosystems may serve as a potential mitigation mechanism to buffer populations against extreme climate events (Roberts et al., 2017; Weiskopf et al., 2020). Moreover, a deeper understanding of these interactions will be crucial for predicting species resilience and sustaining biodiversity in freshwater ecosystems.

### Transgenerational effects of heatwaves on *D. magna*

Understanding the mechanisms underlying transgenerational plasticity in response to heatwave stress is critical for predicting species resilience to climate change. Our results suggest that transgenerational plasticity influences offspring development and reproduction, potentially mediated by parental effects such as epigenetic modifications or altered maternal provisioning. In contrast, *D. magna* exposed to stable warming exhibited a distinct trajectory of life-history traits. Individuals exposed to stable warming had reduced body size at maturity and shorter time to maturation compared with controls, indicating a potential trade-off between thermal tolerance and life-history traits. This trade-off may arise from a physiological shift in energy allocation, as previously suggested (Hoefnagel et al., 2018), where *D. magna* prioritizes survival over development and reproduction under chronic thermal stress. While stable warming facilitated adaptive responses, it also imposed trade-offs that could have long-term ecological consequences (Lancaster et al., 2017).

Our findings highlight the fundamental differences in how *D. magna* responds to heatwaves versus stable warming. While heatwave-induced transgenerational plasticity was partially reversible, with negative effects persisting for up to two generations before recovery, the extent of this plasticity may vary across species, particularly in wild populations experiencing increasingly frequent and intense heatwaves. Species with low adaptive potential and genetic variability, such as endangered taxa, may face an accelerated risk of extinction under frequent heatwave events (Maxwell et al., 2019). Highly heat-sensitive species may struggle to recover from repeated heat stress, leading to long-term population declines (Duffy et al., 2022). Under these circumstances, many species may fail to counteract the cumulative effects of recurring heatwaves, resulting in reduced reproductive success, impaired physiological function and, ultimately, heightened extinction risk.

To explore potential molecular mechanisms underlying these responses, we examined the expression of two vitellogenin genes, *vtg1* and *vtg2*, which play essential roles in yolk production and ovarian maturation (Kato et al., 2004). The increase in *vtg1* expression suggests accelerated somatic growth and reproduction at elevated temperatures (Korsgaard et al., 1986), aligning with our physiological observations of earlier maturation under stable warming. The persistent downregulation of *vtg2* may indicate sensitivity to warming, potentially due to the suppressive effects of ecdysteroids on vitellogenesis in daphnids (Hannas et al., 2011). The recovery of *vtg1* and *vtg2* expression from F2 to F4, consistent with our physiological findings, suggests that *D. magna* gradually overcame the transgenerational effects of heatwave exposure. These distinct expression patterns of *vtg* genes under heatwave and stable warming conditions, along with the observed differential transgenerational effects, provide valuable insights into the regulatory mechanisms of vitellogenesis and reproductive adaptation under thermal stress.

In summary, our study provided evidence of transgenerational effects of heatwaves on *D. magna*, raising concerns about species persistence under intensifying climate extremes. While stable warming facilitated adaptive potential for heat tolerance, it imposed trade-offs that could impact long-term population viability. Future studies should further investigate the genetic and epigenetic mechanisms underlying these differential transgenerational plastic responses. Additionally, linking molecular biomarker changes with physiological responses will be crucial for understanding the mechanisms of heatwave stress in *D. magna* and other aquatic organisms.

### Multigenerational exposure to heatwaves fails to develop heatwave tolerance in *D. magna*

Our multigenerational experiment demonstrated that *D. magna* failed to develop heatwave tolerance and show any beneficial transgenerational plasticity despite repeated heatwave exposure across four clonal generations. The inability to develop beneficial plastic responses implies that episodic heatwaves do not impose adaptive transgenerational effects that could induce heritable adaptations to accumulate over generations. Instead, these transient stressors primarily induce sublethal physiological costs, impairing growth and reproduction and ultimately reducing population viability. This persistent negative transgenerational plasticity contrasts sharply with responses under stable warming conditions, where we observed signs of plasticity, characterized by trade-offs between altered life-history traits and increased heat tolerance. Sustained thermal stress can drive selection for heat-tolerant genotypes, enabling populations to evolve mechanisms that enhance fitness under chronic warming (Howells et al., 2021). The contrast between these scenarios highlights the fundamental difference between immediate phenotypic plasticity and transgenerational plasticity: while plasticity may facilitate short-term physiological adjustments to stable conditions, transgenerational plasticity in response to intermittent stressors appears to be maladaptive (Chen et al., 2022). Although some studies suggest that transgenerational effects may be mediated by epigenetic modifications, such as chromatin remodeling and DNA methylation (Champagne, 2008; Fitz-James and Cavalli, 2022), the molecular mechanisms underlying heatwave-induced transgenerational and multigenerational effects remain poorly understood. However, one of the limitations of this study is that all individuals originated from a single genotype that reproduces clonally, which eliminates confounding effects of standing genetic variation and allows us to isolate non-genetic mechanisms but restricts the potential for selection-driven evolutionary responses, and these results suggest transgenerational plasticity and potential epigenetic responses under multigenerational heatwave exposure. To better understand evolutionary responses to heatwaves, future studies should include sexual reproduction phases to enable genetic recombination and facilitate adaptive evolution through the selection of heat-tolerant genotypes. Additionally, further research should examine these mechanisms across a wider taxonomic range to determine whether diverse species can develop adaptive strategies for heatwave tolerance.

### Conclusions

In the context of climate change, extreme heatwave events are becoming more frequent and intense, subjecting marine and freshwater ecosystems to increasing thermal variability. Using an experimental, multigenerational approach, we investigated both plastic and evolutionary responses to heatwave stress and stable warming in the model zooplankton *D. magna*. Our results show that heatwaves compromise development and reproduction in *D. magna* and that responses to heatwaves are not predictable from those to stable warming. The contrasting life-history responses observed under these two global climate change scenarios highlight their distinct physiological and ecological consequences. Our study further suggests that the impact of heatwaves on *D. magna* is driven by the interplay between warming and cooling phases during heatwaves. We found that the effects of a second heatwave were additive, with reproductive output significantly reduced even after a 5 day acclimation period. Additionally, food availability modulated heatwave responses, with low food levels exacerbating developmental delays and reducing survival. Beyond these direct effects, heatwaves also induced significant transgenerational effects, including reduced body size at maturity, delayed maturation and lower reproductive output in offspring that were never directly exposed to heat stress. By linking molecular biomarker changes with physiological responses, our results reveal differential transgenerational plasticity between heatwave and stable warming treatments in *D. magna*. Under stable warming, we observed a potential trade-off between life-history traits and heat tolerance, suggesting that long-term thermal stress may drive adaptive responses. However, *D. magna* failed to develop heatwave tolerance despite repeated exposure and recovery cycles, indicating that fluctuating heatwave conditions do not promote adaptive plasticity. In contrast, environments with prolonged and consistent warming may favor adaptive responses to thermal stress, potentially enabling populations to persist under novel thermal regimes. These results suggested that even *D. magna* with high evolutionary potential may struggle to cope with the rapid pace and intensity of climate-induced heatwave stress, especially when extreme events occur in succession. Ultimately, the differential impacts of heatwaves and stable warming emphasize the urgent need to consider both types of thermal stress when predicting species' responses to climate change. Future research should investigate whether a broader range of taxa can evolve heatwave tolerance and explore the molecular mechanisms underlying resilience to climate change.

## Supplementary Material

10.1242/jexbio.250837_sup1 Supplementary information
