# Supplementary material for: Complex and lasting impacts of heatwaves on life-history traits and fitness in Daphnia magna
Source: J Exp Biol. 2025 Sep 26;228(18):jeb250837. doi: 10.1242/jeb.250837 (PMC12517341; doi:10.1242/jeb.250837)
Supplement: Supplementary information [file jexbio-228-250837-s1.pdf]

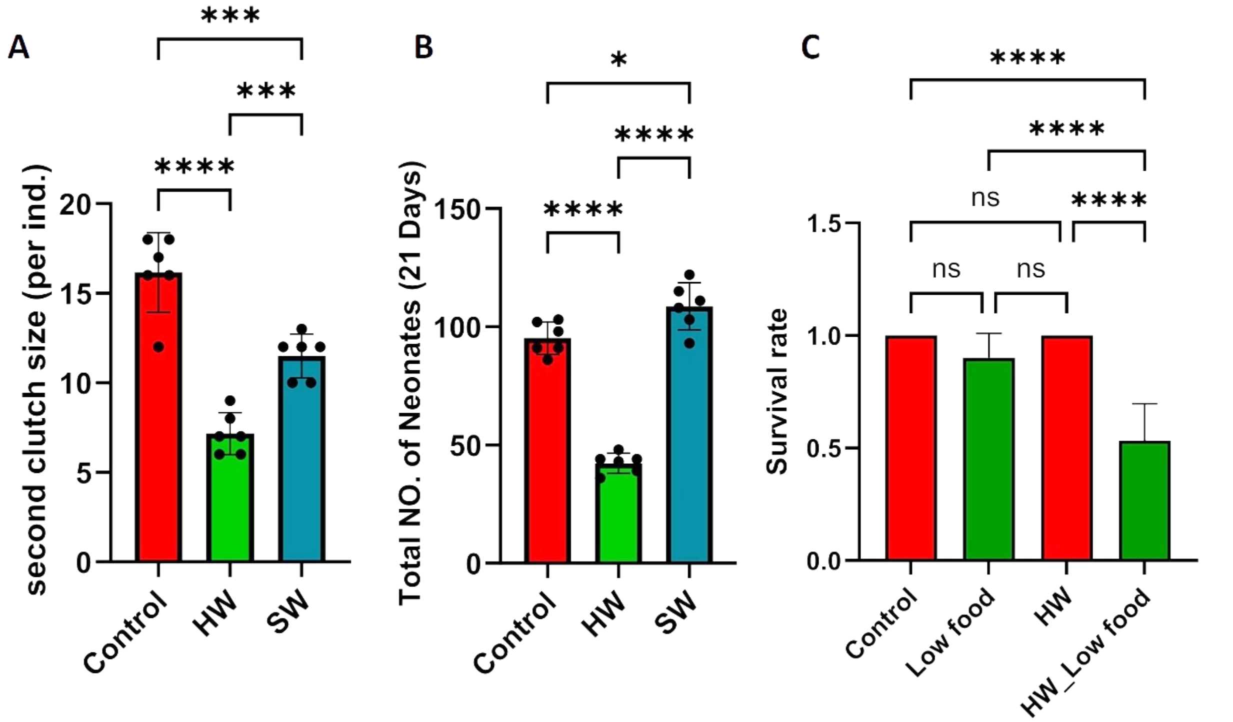

Fig. S1. Heatwave exposure impairs reproduction in *Daphnia magna*. A) second clutch size and B) Total number of offsprings of 21 days for Control, Heatwave (HW) and Stable warming (SW) groups. C) The survival rate of *D. magna* under interactive effects of food limitation and heatwave exposure.

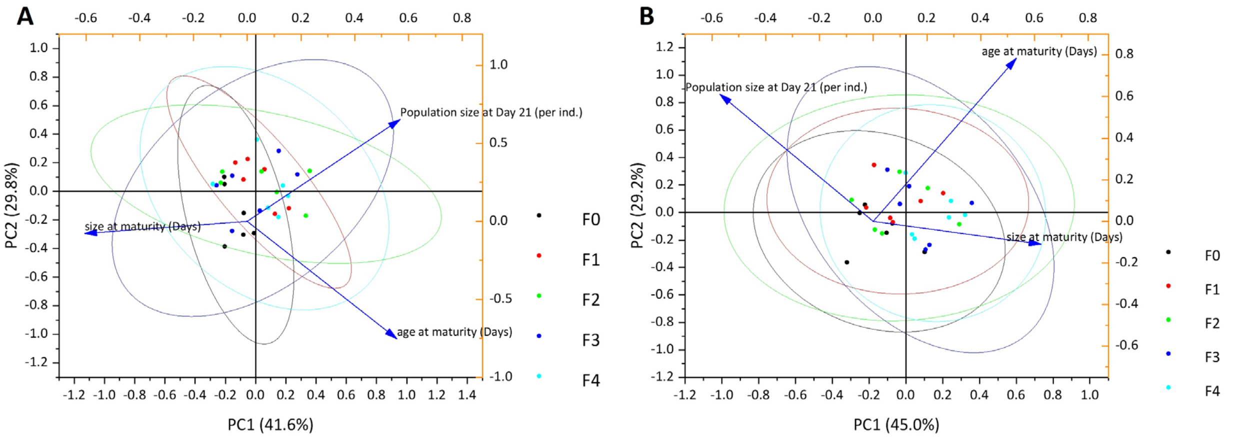

Fig. S2. Principal component analysis (PCA) of life-history traits across six replicates over multiple generations for A) Control and B) Stable warming group.

Journal of Experimental Biology • Supplementary information

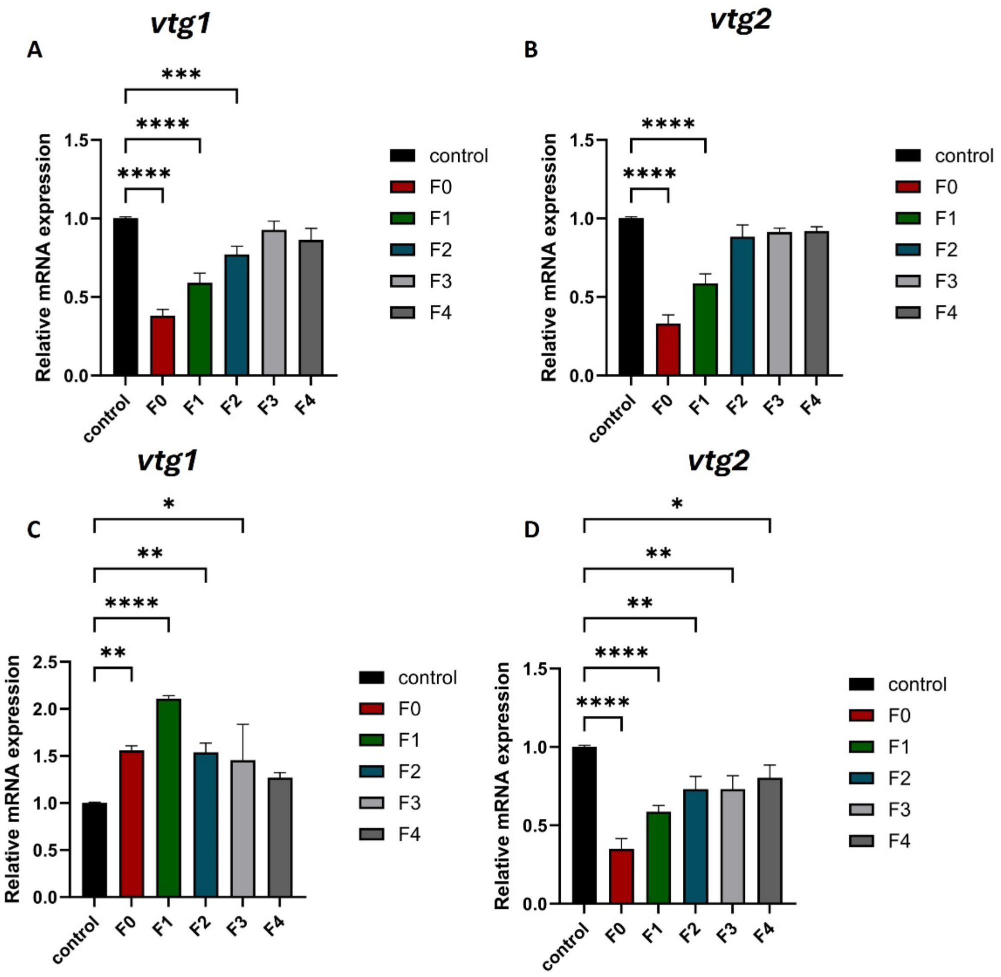

Fig. S3. Expression profiles of vitellogenin genes (*vtg1* and *vtg2*) across generations for A-B) heatwave and C-D) stable warming group.

Journal of Experimental Biology • Supplementary information

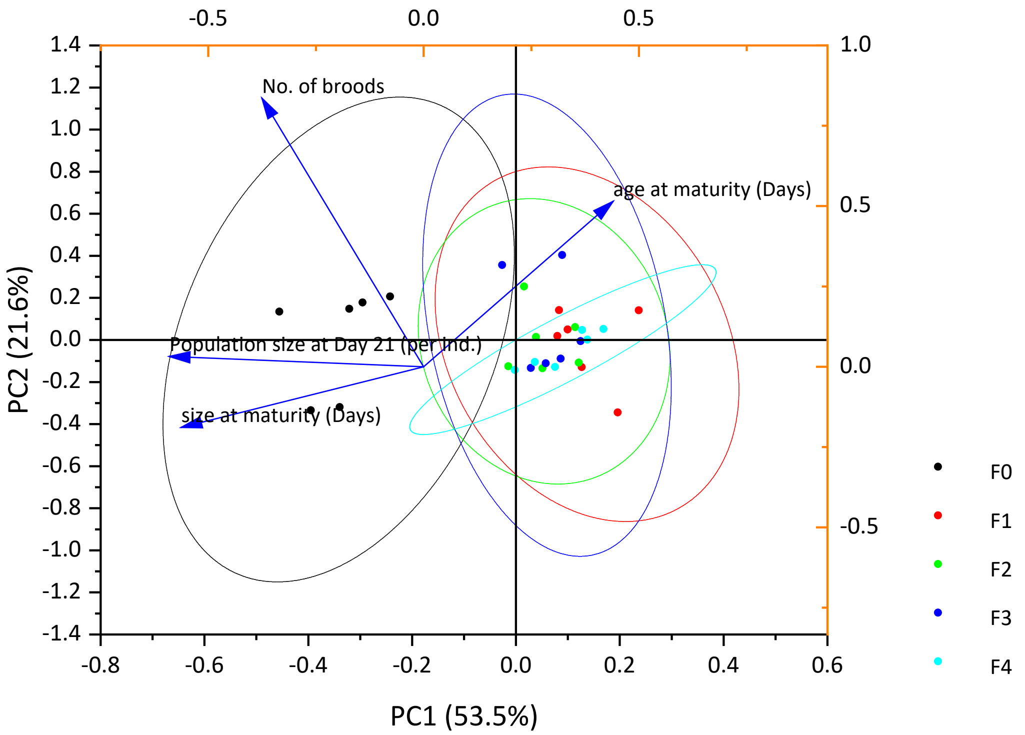

Fig. S4. Principal component analysis (PCA) of life-history traits across generations of control group.

Journal of Experimental Biology • Supplementary information

Table S1. Principal component analysis (PCA) of Life-history traits variables *for each treatment.*

Life-history traits variables	PC1	PC2	PC3
age at maturity (Days)	0.41544	0.3584	0.53471
size at maturity (mm)	-0.21259	0.79688	-0.4483
first clutch size (per ind.)	-0.40689	0.3878	0.63074
Population size at Day 21 (per ind.)	-0.45777	-0.11519	0.02665
Somatic growth rate (Growth/Day)	-0.46301	-0.0157	-0.17163
Time to first reproduction	0.439	0.2695	-0.29173

Table S2. Principal component analysis (PCA) of Life-history traits variables for each generation.

Life-history traits variables	PC1	PC2	PC3
age at maturity (Days)	0.58032	0.55858	-0.59263
Population size at Day 21 (per ind.)	-0.61838	-0.17129	-0.76699
size at maturity (mm)	-0.52994	0.81157	0.24601

Table S3. Principal component analysis (PCA) of Life-history traits variables for each generation.

Life-history traits variables	PC1	PC2	PC3
age at maturity (Days)	-0.6571	-0.18698	-0.36016
Population size at Day 21 (per ind.)	0.68442	0.15226	-0.07764
No. of broods	0.0393	-0.73637	0.64781
size at maturity (Days)	-0.31342	0.63215	0.66678

Journal of Experimental Biology • Supplementary information
